# MicroRNA-10b overexpression promotes non-small cell lung cancer cell proliferation and invasion

**DOI:** 10.1186/2047-783X-18-41

**Published:** 2013-11-12

**Authors:** Yi Liu, Minghui Li, Guoqing Zhang, Zuoliang Pang

**Affiliations:** 1 Department of Thoracic Surgery, Affiliated Tumor Hospital, Xinjiang Medical University, Urumqi, Xinjiang 830011, China

**Keywords:** microRNA-10b, Non-small cell lung cancer, Proliferation, Invasion

## Abstract

**Background:**

miRNAs are a class of small non-coding RNA molecules that play an important role in the pathogenesis of human diseases through negative regulation of gene expression. Although miRNA-10b (miR-10b) has been implicated in other tumors, its role in non-small cell lung cancer (NSCLC) is still unknown. The aim of the present study was to investigate the role of miR-10b in NSCLC.

**Methods:**

Expression of miR-10b was analyzed in NSCLC cell line A549 by qRT-PCR. Cell viability was evaluated using Cell Counting Kit (CCK)-8. Cell migration and invasion were evaluated by wound healing assay and transwell assays. Cell cycle and apoptosis analyses were performed. Western blotting was used to predicate the target of miR-10b.

**Results:**

The A549 cell line transfected with the miR-10b exhibited significantly increased proliferation, migration, and invasion capacities when compared with the control cells (*P* < 0.05). Krüppel-like factor 4 (KLF4) may be indirectly targeted by miR-10b during the proliferation increasing of A549 cells.

**Conclusion:**

In this study, we found that miR-10b is a tumor enhancer in NSCLC. Thus, miR-10b may represent a potential therapeutic target for NSCLC intervention.

## Background

Non-small cell lung cancer (NSCLC) is the predominant form of lung cancer, and accounts for the majority of cancer deaths worldwide [1]. The prognosis of lung cancer is still unfavorable, with a 5-year overall survival rate of approximately 11%, despite recent advances in clinical and experimental oncology [2]. Thus, detailed NSCLC development and progression research is essential for improving the diagnosis, prevention, and treatment of this disease. Recently, an increasing number of reports have shown that non-coding small RNAs may be involved in NSCLC pathogenesis, providing new insights into disease biology.

The miRNAs are endogenous small non-coding RNAs of 21 to 24 nucleotides that regulate gene expression by base pairing with target mRNAs in the 3′-untranslated region (3′-UTR), leading to mRNA cleavage or translational repression [3,4]. There are now more than 700 human miRNAs annotated in the miRBase database (University of Manchester, Manchester, UK), and it has been predicted that in total there are more than 1,000 human miRNAs [5,6]. It is estimated that approximately one third to one half of human genes are regulated by miRNAs, and each miRNA is predicted to target several hundred transcripts, making miRNAs one of the largest families of gene regulators. Dysregulation of miRNAs may lead to alterations in cellular differentiation, proliferation, and apoptotic processes of cancer [7,8]. Indeed, deregulation of miRNAs is closely associated with tumor initiation, promotion, and progression through the regulation of key oncogenes or tumor suppressors [9-13]. Thus, understanding the biological consequences of miRNA dysregulation and identifying miRNA targets are critical for diagnosis, prevention, and treatment of cancer.

miRNA-10b (miR-10b) has been reported to play a role in the invasion and metastasis of cancer. miR-10b is highly expressed in metastatic breast cancer cell lines and metastatic breast tumors of patients. miR-10b upregulation inhibits the translation of HOXD10, a transcription factor known for its roles in cell motility, leading to the increasing expression of the prometastatic gene *RhoC*[14]. Silencing of miR-10b significantly decreases miR-10b levels and increases the levels of HOXD10 to inhibit metastasis [15]. MiR-10b expression has subsequently been shown to correlate with the migration and invasion of human esophageal cancer cell lines through regulation of Kruppel-like factor 4 (KLF4) expression [16]. Moreover, miR-10b is overexpressed in malignant glioma, pancreatic cancer, and nasopharyngeal carcinoma [17-20]. However, whether overexpression of miR-10b is involved or not in NSCLC pathogenesis has not yet been investigated.

In this study, we focused on the expression and roles of miR-10b in the A549 cell line, and found that miR-10b promotes the proliferation and invasion of A549 cells *in vitro*.

## Methods

### Cell lines and culture conditions

NSCLC adenocarcinoma cell lines A549 and H1299 were purchased from the Institute of Biochemistry and Cell Biology of the Chinese Academy of Sciences (Shanghai, China). Cells were cultured in RPMI 1640 or medium supplemented with 10% FBS (Gibco, Carlsbad, CA, USA), 100 U/mL penicillin, and 100 mg/mL streptomycin (Invitrogen, Carlsbad, CA, USA) in humidified air at 37°C with 5% CO_2_.

### Cell transfection

The miR-10b (sense: 5′-UAC CCU GUA GAA CCG AAU UUG UG-3′, antisense: 3′-CAA AUU CGG UUC UAC AGG GUA UU-5′) eukaryotic expression plasmid was obtained from GenePharma Company (Shanghai, China). An antisense (anti-miR-10b; sequence: 5′-CAC AAA UUC GGU UCU ACA GGG UA-3′) was used to inhibit miR-10b expression. A scrambled RNA duplex (sense: 5′-UUC UCC GAA CGU GUC ACG UTT-3′, antisense: 5′-ACG UGA CAC GUU CGG AGA ATT-3′) expression plasmid was used as the control. The day before transfection, A549 cells were seeded in antibiotic-free medium. Transfections were carried out using Lipofectamine 2000 (Invitrogen) in accordance with the manufacturer’s instructions. To monitor transfection efficiency, successfully transfected cells were observed with a fluorescence microscope.

### qRT-PCR

Total RNA, including miRNA, was isolated with TRIzol Reagent (Invitrogen) according to the manufacturer’s instructions. Expression of hsa-miR-10b was analyzed with the miScript system (Qiagen, Venlo, The Netherlands), which consists of the miScript RT Kit, miScript Primer Assay, and miScript SYBR Green PCR Kit, according to the protocol provided by the company. Small nuclear RNA U6 was used for normalization; cDNA was synthesized using Moloney Murine Leukemia Virus Reverse Transcriptase (Takara, Dalian, China) as described by the manufacturer. Primers used were as follows: miR-10b forward primer: 5′- GGA TAC CCT GTA GAA CCG AA -3′ and reverse primer: 5′-CAG TGC GTG TCG TGG AGT-3′. U6 sense: 5′-CTC GCT TCG GCA GCA CA-3′ and reverse: 5′-AAC GCT TCA CGA ATT TGC GT-3′. The PCR reaction was conducted at 95°C for 30 seconds, followed by 40 cycles of 95°C for 5 seconds and 60°C for 34 seconds on the ABI Prism 7700 Sequence Detector (Applied Biosystems, Carlsbad, CA, USA). All of the reactions were run in triplicate. The comparative method (∆∆C_T_) was used for relative quantification of gene expression to determine miR-10b mRNA expression levels.

### Wound healing assay

Cells transfected with miR-10b, negative control, and anti-miR-10b were plated in 35 mm dishes. When cells grew to confluence, a line was traced with a pipette tip. A549 cells were then washed with serum-free medium and incubated with RPMI 1640. The wound was photographed at 0, 24, 48, and 72 hours.

### Annexin-V-fluorescein isothiocyanate (FITC) apoptosis assay

Cells were collected after miR-10b transfection for 72 hours, and the translocation of phosphatidylserine in treated cells was detected using the Annexin-V-FLUOS Staining Kit (Roche Applied Science, Mannheim, Germany). Briefly, cells were suspended in 500 mL of binding buffer and incubated at room temperature in the dark for 15 minutes after being labeled with 5 mL of Annexin-V-fluorescein isothiocyanate (FITC) and 5 mL of propidium iodide (PI). The stained cells were then analyzed by flow cytometry.

### Cell proliferation assay and cell cycle analysis

Cell viability was assessed by Cell Counting Kit (CCK)-8 kit (Tongren, Shanghai, China). Briefly, 0.5 × 10^4^ cells were seeded in each 96-well plate, transfected with the indicated miR-10b, and further incubated for 24, 48, and 72 hours, respectively. Approximately 10 mL CCK-8 reagent was added to each well at 1 hour before the endpoint of incubation. The optical density (OD) 450 nm values in each well were determined by a microplate reader.

For cell cycle analysis, cells were fixed, stained with PI, and examined with a fluorescence-activated cell sorting (FACS) flow cytometer (Beckman Coulter, Pasadena, CA, USA). DNA histograms were analyzed using MultiCycle software (Phoneix Flow Systems, San Diego, CA, USA).

### Invasion assay

Cell invasion was analyzed with uncoated transwell cell culture chambers (8 μm pore size) (Yu Kangning, **Shanghai,** China). Briefly, 48 hours after transfection, cells were resuspended in serum-free medium, and 200 μL of the cell suspension (4 × 10^4^ cells) was added to the upper chamber. Medium containing 10% serum as a chemoattractant was added to the bottom wells of the 24-well chamber. For the screen, the cells that did not invade after 24 hours were removed from the upper face of the filters by scrubbing with a cotton swab, after which the membrane was fixed with 4% formaldehyde for 10 minutes at room temperature and stained with 0.5% crystal violet for 10 minutes. Finally, invasion cells were counted from ten different fields of each filter. Experiments were repeated in triplicate.

### Western blot analysis

Immunoblotting was performed to detect the expression of KLF4 in A549 cell lines. Cultured or transfected cells were lysed in radioimmunoprecipitation assay (RIPA) buffer with 1% phenylmethanesulfonyl fluoride (PMSF). Protein was loaded onto an SDS-PAGE mini-gel and transferred onto polyvinylidene difluoride (PVDF) membrane. The blots were probed with 1:1,000 diluted rabbit polyclonal KLF4 antibody (Abcam, Cambridge, UK) at 4°C overnight and subsequently incubated with horseradish peroxidase (HRP)-conjugated secondary antibody (1:5,000). Signals were visualized using ECL Substrates (Millipore, Billerica, MA, USA). GAPDH was used as an endogenous protein for normalization.

### Statistical analysis

All data from three independent experiments were expressed as mean ± SD and processed using SPSS 17.0 statistical software (IBM, Armonk, NY, USA). The difference was estimated by Student’s *t*-test or one-way ANOVA. A value of *P* <0.05 was considered to be statistically significant.

## Results

### miR-10b expression in A549 cells

**Figure 1:**
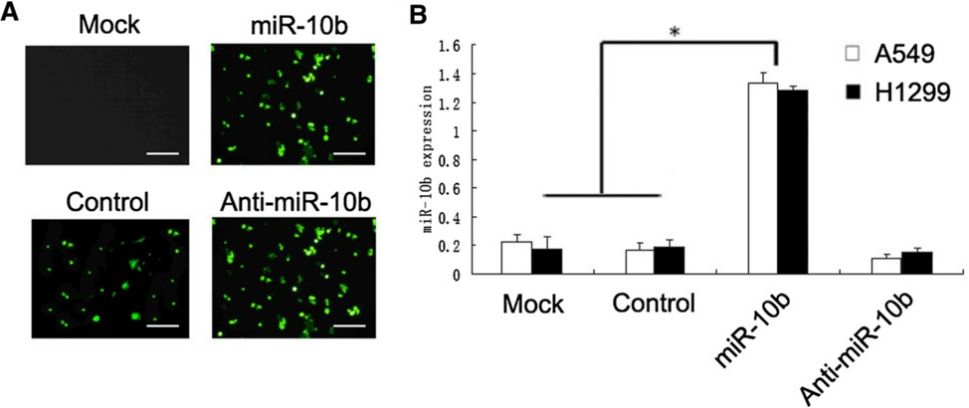
**miR-10b expression on A549 and H1299 cells. (A)** Fluorescence photomicrographs of A549 cells infected by miR-10b at a multiplicity of transfection of 10. Images were taken 72 hours after infection. Scare bar = 100 μm. **(B)** miR-10b was subjected to real-time PCR analysis for miR-10b and U6 expression levels. Ectopic expression of miR-10b on A549 and H1299 cells was analyzed by qRT-PCR. Asterisks indicate significant difference when compared with control (*P* <0.05). miR-10b, microRNA-10b.

To further study the biological role of miR-10b in lung cancer progression, we transfected A549 and H1299 cells with GFP-labeled plasmids carrying miR-10b. We routinely observed a highly efficient infection (> 90%) 72 hours after transfection at multiplicity of transfection of 10 through estimating EGFP expression under a fluorescent microscope (Figure 1A). We performed SYBR green quantitative PCR analysis to detect the expression levels of miR-10b in A549 and H1299 cells. The level of miR-10b expression in cultures of A549 cells was greater than the control group (Figure 1B).

### miR-10b inhibition leads to increase of A549 cell growth

**Figure 2:**
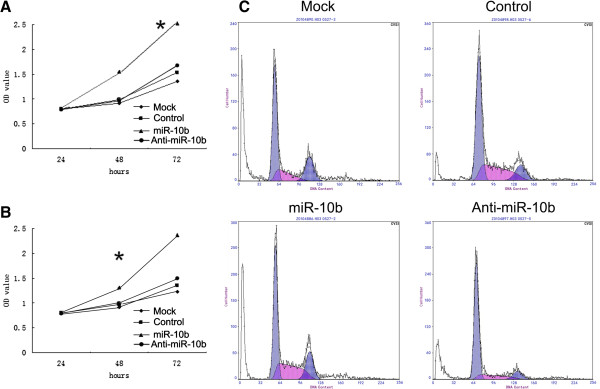
**miR-10b promotes proliferation of A549 and H1299 cell lines. (A)** A549 cell proliferation significantly increased after miR-10b expression. Asterisks indicate significance compared with control (*P* <0.05). **(B)** H1299 cell proliferation significantly increased after miR-10b expression. Asterisks indicate significance compared with control (*P* <0.05). **(C)** Cell cycle distributions of A549 cells transfected with the miR-10b. miR-10b, microRNA-10b.

Understanding the regulation of cell proliferation will be critical for the development of new and more successful therapies for preventing and treating cancer, and for the screening of new anticancer drugs. Therefore, the rapid and accurate assessment of cell proliferation is an important requirement in many experimental situations involving *in vitro* and *in vivo* studies. Viability assays were used as a further study to investigate the effect of miR-10b on proliferation of A549 cells. The results of this assay showed that miR-10b could enhance the A549 cell growth remarkably (Figure 2A) and also in H1299 cells (Figure 2B), and provides evidence that miR-10b plays a key role in promoting the development of lung cancer.

To test whether miR-10b affected the behavior of A549 cells, we detected the cell cycle of A549 cells with miR-10b or anti-miR-10b transfection. Compared with the cells transfected with the negative control miRNA, the proliferation of A549 cells transfected with anti-miR-10b was significantly decreased (Figure 2C), while A549 cell growth increased in miR-10b transfection, indicating that miR-10b may increase A549 cell proliferation.

### MiR-10b promotes the invasiveness of A549 cells

**Figure 3:**
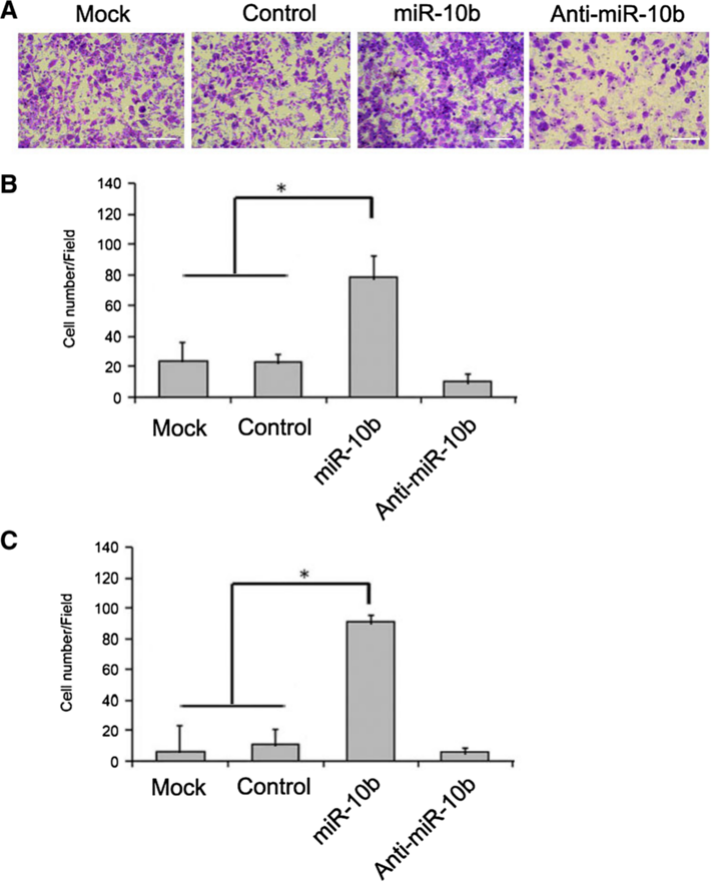
**miR-10b suppresses the invasion of A549 and H1299 cells. (A)** A549 cells transfected with the miR-10b or inhibitor were subjected to Matrigel migration assays. The migrated cells were stained with crystal violet for 30 minutes. Scare bar = 100 μm. **(B)** A549 cells were counted and analyzed. Data shown are mean ± SD of triplicate measurements. *P <0.05. **(C)** H1299 cell migration assays were performed. Data shown are mean ± SD of triplicate measurements. *P <0.05. miR-10b, microRNA-10b.

We investigated the role of miR-10b in the invasiveness of A549 and H1299 cells, which is an important aspect of malignant progression and metastasis. MiR-10b was significantly overexpressed after transfection of the miR-10b. As shown in Figure 3, the number of invading A549 and H1299 cells transfected with the miR-10b was greater than the control group (*P* < 0.05). These findings suggest that miR-10b expression may play a specific role in the invasiveness of A549 cells.

**Figure 4:**
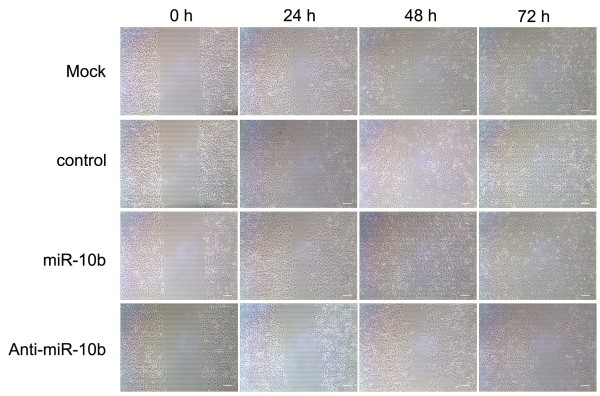
**miR-10b promotes the migration and invasion of A549 cells.** A549 cells were transfected with miR-10b or inhibitor. Cells were subjected to wound healing assays and images were taken at 0, 24, 48, and 72 hours. Scare bar = 100 μm. miR-10b, microRNA-10b.

We further investigated the effects of miR-10b on A549 cell migration, two essential steps for malignant progression and metastasis. A549 cells transfected with the miR-10b or anti-miR-10b were applied to wound healing assays. The results showed that miR-10b significantly increased the migration of A549 cells, whereas anti-miR-10b decreased the migration of A549 cells (Figure 4).

### Without induction of A549 cell apoptosis by miR-10b

**Figure 5:**
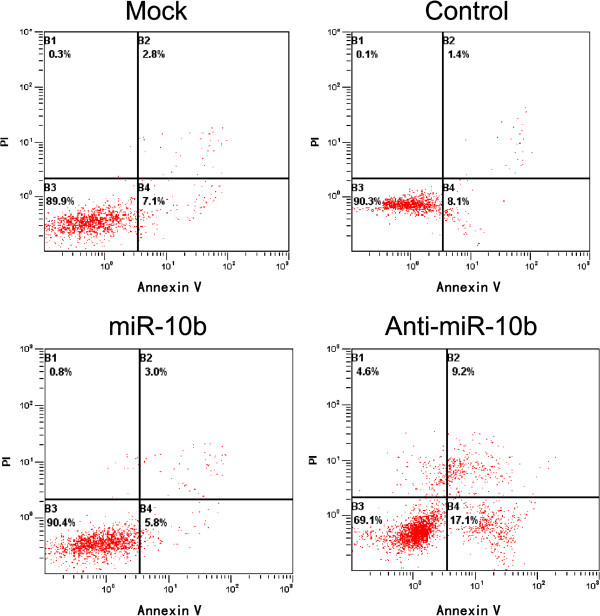
**Apoptosis assay.** After transfection with miR-10b overexpression vector for 48 hours, Annexin-V-FITC and PI co-staining were used for flow cytometric analysis. FITC, fluorescein isothiocyanate; miR-10b, microRNA-10b; PI, propidium iodide.

To further confirm the important role of miR-10b in A549 cells, we examined the effects of miR-10b overexpression on apoptosis induced by this miRNA. After successful transfection of miR-10b and anti-miR-10b, we found that overexpression of miR-10b did not significantly inhibit the early and late apoptosis, and total apoptotic cell death in A549 cells (Figure 5). These results suggest that overexpression of miR-10b could promote A549 cell survival.

### Examination of the targeting of miR-10b

**Figure 6:**
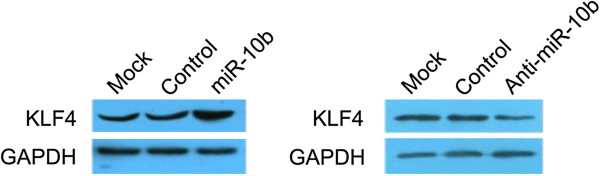
**Prediction of miR-10b target gene.** KLF4 protein levels were detected by Western blot assay. The expression of KLF4 was increased by miR-10b when compared with the control and anti-miR-10b. KLF4, Krüppel-like factor 4; miR-10b, microRNA-10b.

Western blot analysis showed that the KLF4 protein expression levels were significantly increased after miR-10b transfection; in contrast, the anti-miR-10b transfection effectively downregulated the KLF4 protein expression levels as examined by western blot analysis (Figure 6), which indicated that the *KLF4* gene was not the indirect target of miR-10b.

## Discussion

In recent years, molecular genetic studies have shown that abnormalities in non-coding miRNAs represent the most recent class of epigenetic mechanisms, which can also contribute to the different steps of tumor development by regulating genes that control cellular processes such as the cell cycle and apoptosis. Recently, several studies have shed light on the importance of miRNAs in cancer invasion and metastasis. Upregulation of miR-10b expression has been detected in metastatic breast cancer, esophageal cancer, malignant glioma, pancreatic cancer, and others [21-24], and miR-10b can positively regulate migration and invasion in both breast cancer and esophageal cancer cell lines [25-29]. Moreover, miR-10b also has a role in regulating angiogenesis in gliomagenesis [20]. However, the biological consequences of miR-10b overexpression have not been characterized in lung cancer. In this study, we investigated the role and functional target of miR-10b in A549 cells.

We showed that transfected miR-10b was associated with growth promotion and invasiveness of A549 cells, and these findings are consistent with a previous study. Ma *et al*. showed that miR-10b decreased the expression of HOXD10, resulting in increased expression of RhoC [9]. In addition, Sasayama *et al*. showed an association between miR-10b and uPAR [15], which is considered to be one of the downstream targets of HOXD10. We did not detect HOXD10 expression in lung cancer, and there have been no previous reports regarding HOXD10 expression and lung cancer. Therefore, there may be no relationship between HOXD10 and uPAR/RhoC expression in lung cancer, although uPAR and RhoC were reported to be correlated with the invasiveness of lung cancer. Identification of putative miRNA targets is important for a complete understanding of the specific functions of miRNAs. In this study, we identified KLF4 as an indirect target of miR-10b, and demonstrated that upregulation of miR-110b significantly increases KLF4 expression at protein level in NSCLC cells. Other mechanisms may affect the invasiveness of lung cancer cells, but since only functional analysis was studied, further investigation such as loss of function of miR-10b will be needed in the future.

## Conclusions

In conclusion, we found that miR-10b is a tumor enhancer in NSCLC. Thus, miR-10b may represent a potential therapeutic target for NSCLC intervention.

## Abbreviations

3′-UTR: 3′-untranslated region; ANOVA: Analysis of variance; CCK: Cell counting kit; EGFP: Enhanced green fluorescent protein; FBS: Fetal bovine serum; FITC: Fluorescein isothiocyanate; FACS: Fluorescence-activated cell sorting; GAPDH: Glyceraldehyde 3-phosphate dehydrogenase; GFP: Green fluorescent protein; HOXD10: Homeobox D10; HRP: Horseradish peroxidase; KLF4: Krüppel-like factor 4; miRNA: microRNA; miR-10b: microRNA-10b; NSCLC: Non-small cell lung cancer; OD: Optical density; PMSF: Phenylmethanesulfonyl fluoride; PCR: Polymerase chain reaction; PVDF: Polyvinylidene difluoride; PI: Propidium iodide; qRT-PCR: quantitative reverse transcription polymerase chain reaction; RIBA: Radioimmunoprecipitation assay; RhoC: Ras homolog gene family member C; RT: Reverse transcription; RPMI: Roswell Park Memorial Institute; uPAR: urokinase-type plasminogen activator receptor.

## Competing interests

The authors declare that they have no competing interests.

## Authors’ contributions

YL, LMH, PZL, and GQZ conceived and designed the study, and analyzed the data. YL and GQZ wrote the paper. All authors read and approved the final manuscript.
